# Tracking causal pathways in TMS-evoked brain responses

**DOI:** 10.1371/journal.pcbi.1013316

**Published:** 2025-07-28

**Authors:** Jinming Xiao, Qing Yin, Lei Li, Yao Meng, Xiaobo Liu, Wanrou Hu, Xinyue Huang, Yu Feng, Xiaolong Shan, Weixing Zhao, Peng Wang, Xiaotian Wang, Youyi Li, Huafu Chen, Xujun Duan

**Affiliations:** 1 Sichuan Provincial Center for Mental Health, Sichuan Provincial People’s Hospital, School of Life Science and Technology, University of Electronic Science and Technology of China, Chengdu, PR China; 2 MOE Key Lab for Neuro information, High-Field Magnetic Resonance Brain Imaging Key Laboratory of Sichuan Province, University of Electronic Science and Technology of China, Chengdu, PR China; 3 McConnell Brain Imaging Centre, Montreal Neurological Institute, McGill University, Montreal, Canada; University of Nottingham, UNITED KINGDOM OF GREAT BRITAIN AND NORTHERN IRELAND

## Abstract

Exploring how local perturbations of cortical activity propagate across the brain network not only helps us understanding causal mechanisms of brain networks, but also offers a network insight into neurobiological mechanisms for transcranial magnetic stimulation (TMS) treatment response. The concurrent combination of TMS and electroencephalography (EEG) enables researchers to track the TMS-evoked activity, defined here as scalp-recorded electrical signals reflecting the brain’s response to TMS, with millisecond-level temporal resolution. Based on this technique, we proposed a quantitative framework which combined sparse non-negative matrix factorization and stage-dependent effective connectivity methods to infer the causal pathways in TMS-evoked brain responses. We found that single-pulse TMS firstly induces local activity in the directly stimulated regions (left primary motor cortex, M1), and then propagates to the contralateral hemisphere and other brain regions. Finally, it propagates back from the contralateral region (right M1) to the stimulation region (left M1). This study provides preliminary evidence demonstrating how local perturbations propagate through brain networks to influence various cortical regions, and offers insights into the neural mechanism of TMS-evoked brain responses from a network perspective.

## Author summary

The concurrent combination of TMS and EEG offers a powerful intervention paradigm for uncovering the causal mechanisms of brain networks. By delivering single-pulse stimulation to a specific brain region and recording the resulting electrical signal using EEG, this technique actively perturbs the system and captures the immediate causal effects of stimulation. TMS-EEG directly tests causal hypotheses, providing evidence of how activity in a stimulated region influences downstream pathways. In this study, we proposed a quantitative framework which combined sparse non-negative matrix factorization and stage-dependent effective connectivity methods to infer the causal pathways in TMS-evoked brain responses. We found that single-pulse TMS firstly induces local activity in the directly stimulated regions (left M1), and then propagates to the contralateral hemisphere and other brain regions. Finally, it propagates back from the contralateral region (right M1) to the stimulation region (left M1). This study provides a preliminary exploration of how local perturbation influence brain network dynamics, and offers insights into the neural mechanism of precise TMS intervention from a network perspective.

## Introduction

Understanding how causal interactions emerge and propagate within brain networks is fundamental to deciphering how distributed neural systems support cognition and behavior [1–4]. The cognitive processes of the human brain involve both bottom-up and top-down mechanisms, which closely correspond to feedforward and feedback information flow within brain networks. Feedforward pathways transmit sensory-driven signals from lower- to higher-order regions, while feedback pathways convey top-down influences such as attention, expectation, and prediction [5,6]. This directional causal flow is fundamental to hierarchical information processing and cognitive control in the brain. To estimate causal interactions between brain regions, researchers have developed effective connectivity techniques to infer the direction and strength of information flow based on neuroimaging data. effective connectivity reflects model-based estimations of how activity in one region statistically influences another under specific assumptions [7]. For instance, Granger Causality (GC) identifies directional dependencies by evaluating whether one brain region’s activity improves the prediction of another region’s future activity [8–10]; Dynamic causal modeling (DCM) incorporates biophysical models to infer casual direction by estimating the directed influence of one neural population on another [11–13]. Phase Slope Index (PSI) measures the direction of information flow by evaluating the frequency-dependent phase differences between signals, identifying consistent phase delays that indicate the leading and lagging relationships between brain regions [14,15]. Recent investigations into causal interactions within the brain predominantly utilize observational data such as resting-state or task-evoked recordings, in combination with effective connectivity frameworks, to infer directed information flow among distributed brain regions. According to the principles of causal inference, paradigms based on observational data are fundamentally limited in their capacity to uncover true causal directionality. Although such paradigms can identify statistical dependencies between brain regions, they offer limited control over confounding variables and are insufficient for establishing definitive causal relationships [16,17]. Intervention-based paradigms involve actively manipulating of the proposed “cause” and evaluating its effects under varying conditions on the “effect”, offering a more robust framework for inferring true causal relationships [4,18]. The concurrent TMS-EEG technique offers a unique opportunity to transition to this interventionist paradigm [19–21]. By applying single-pulse stimulation to a targeted cortical region and simultaneously recording neural responses via EEG, this approach enables researchers to actively perturb the system and observe its causal influence on distributed brain activity in real time. Unlike approaches based solely on observational data, TMS-EEG permits direct testing of causal hypotheses, yielding clear evidence of how activity in a stimulated region affects functionally connected downstream areas [21].

The combination of concurrent TMS-EEG and effective connectivity techniques for exploring causal brain networks has gained increasing attention in recent years. Ye et al. indicated that information-theoretic approach can detect directional information flow of TMS-evoked brain activity [22]. Bevilacqua et al. applied repeated paired single-pulse TMS over primary visual cortex and medial temporal area regions and utilized Granger Causality analysis to investigate the directional information flow between these regions [23]. Moreover, TMS-EEG has been used to investigate the abnormal connectivity in neurological disorders such as disorders of stroke [24]. However, it is important to note that these previous studies have primarily relied on time-cumulative measures of effective connectivity, estimating directional interactions based on the whole post-stimulation time window (typically several hundred milliseconds). While this approach is effective for detecting causal influences in relatively simple, unidirectional propagation scenarios, it lacks the resolution needed to capture the full spatial-temporal complexity of neural dynamics, particularly when both feedforward and feedback processes are involved. Especially, by using source-localized TMS-EEG analysis, which reconstructs EEG signals from sensor space to cortical sources to allow anatomically specific interpretation of neural responses, and whole-brain connectome-based computational modelling, which simulates brain dynamics based on structural connectivity across the entire cortex, Momi et al indicated that the initial EEG signal changes was caused by local dynamics in stimulation regions, while later EEG signal changes of stimulation regions were influenced by activity within a wider connected network [25]. These results indicated that TMS-evoked brain activity involved a recurrent, re-entrant propagation pattern, wherein activity initially spread from the stimulation site to distributed cortical regions and subsequently re-converged upon the original stimulation site, reflecting large-scale feedback dynamics. This study supports the notion that TMS-evoked activity propagation is inherently dynamic and unfolds across distinct temporal stages, encompassing early local responses and later re-entrant dynamics. This stage-dependent structure, which we defined in our methodological framework, underscoring the necessity of employing dynamic effective connectivity analysis to achieve a more detailed and accurate understanding of the causal pathways involved in TMS-evoked brain responses.

This study attempts to establish a quantitative framework for investigating the comprehensive causal pathways in TMS-evoked brain responses. Firstly, to disentangle the complex spatiotemporal structure of TMS-evoked responses, we employed sparse non-negative matrix factorization (sNMF) to decompose the source-reconstructed EEG data into a low-dimensional representation comprising spatial and temporal components. Specifically, the spatial components—referred to as co-activation modules—capture patterns of cortical regions that tend to be concurrently activated following TMS stimulation. The associated temporal components—time-varying weights—track the relative contribution or activation strength of each co-activation module across time, thereby capturing the evolving dynamics of TMS-evoked responses. [26–28]. Secondly, we adopted a hierarchical temporal framework that comprising fine-grained “states” and coarser-grained “propagation stages”. In this framework, states represent short, temporally stable configurations of cortical activity, while propagation stages integrate multiple adjacent states to reflect broader periods of activity propagation. We conducted PSI analysis for each propagation stages to capture the dynamic propagation patterns of TMS-evoked brain activity. The primary aim of this study is to provide a preliminary exploration of how local perturbations influence brain network dynamics, offering insights into the comprehensive causal pathways of TMS-evoked brain activity.

## Results

### The spatiotemporal structure of TMS-evoked brain activity

**Fig 1:**
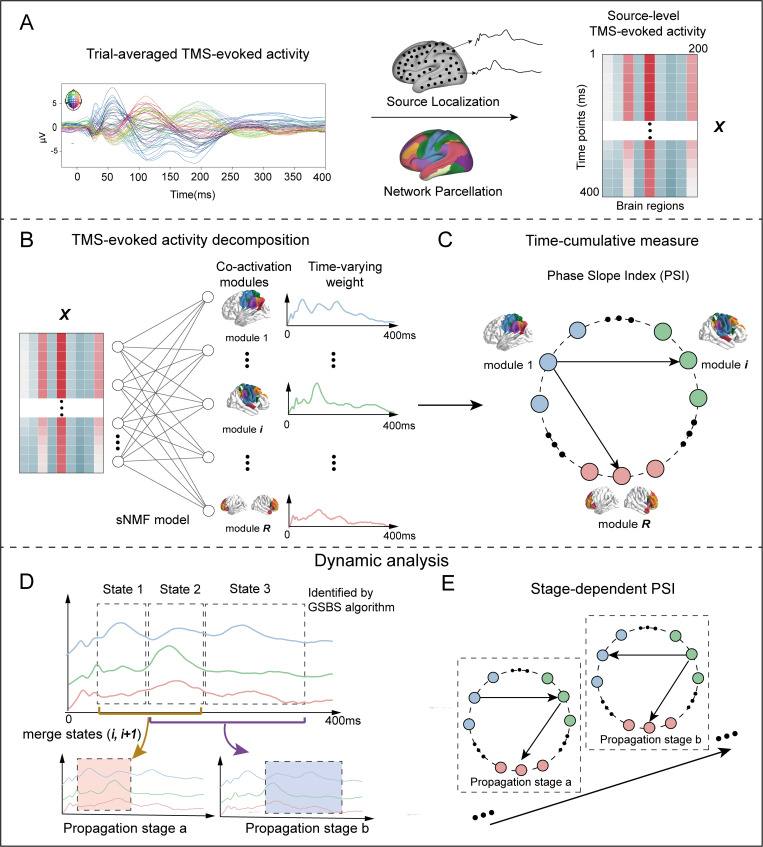
Methodological overview. (A) The source reconstruction of TMS-evoked potential of each subject was performed using dSPM method based on MNE software library. The time series of cortical activity were extracted through Schaefer 200 parcellation atlas. The left panel illustrated the averaged sensory-level TMS-evoked potential waveforms. (B) By applying the Sparse nonnegative matrix factorization (sNMF) method, the TMS-evoked activity was decomposed into co-activation modules, representing spatially specific network components, and time-varying weights, representing temporal expressions of modules. (C) Time-cumulative measure of TMS-evoked activity propagation was performed by calculating the phase slope index (PSI) between different co-activation modules throughout the entire duration (0~400ms following stimulation). To capture the dynamic propagation patterns of TMS-evoked brain activity, (D) we adopted a hierarchical temporal framework comprising fine-grained states and coarser-grained propagation stages. In this framework, states refer to temporally stable configurations of cortical activity, identified using the Greedy State Boundary Search (GSBS) algorithm. Adjacent states were subsequently merged into broader propagation stages to reflect distinct stages of TMS-evoked propagation. (E) Within each propagation stage, we computed the Phase Slope Index (PSI) between co-activation modules, defining this as stage-dependent PSI. This strategy enabled us to characterize the dynamic propagation patterns of TMS-evoked brain activity. All elements in this figure were created by the authors using hand-drawing and open-source Python software.

To unravel the complex spatiotemporal structure of TMS-evoked brain activity, we applied the sparse nonnegative matrix factorization (sNMF) method to decomposed the TMS-evoked brain activity (0~400ms following stimulation) into 10 co-activation modules and corresponding time-varying weights (Fig 1A and 1B). This decomposition was performed using optimal parameters (R=10 for the number of modules, and α=20% for the sparsity threshold). For detailed information regarding parameter optimization, please refer to Fig A in S1 Text.

**Fig 2:**
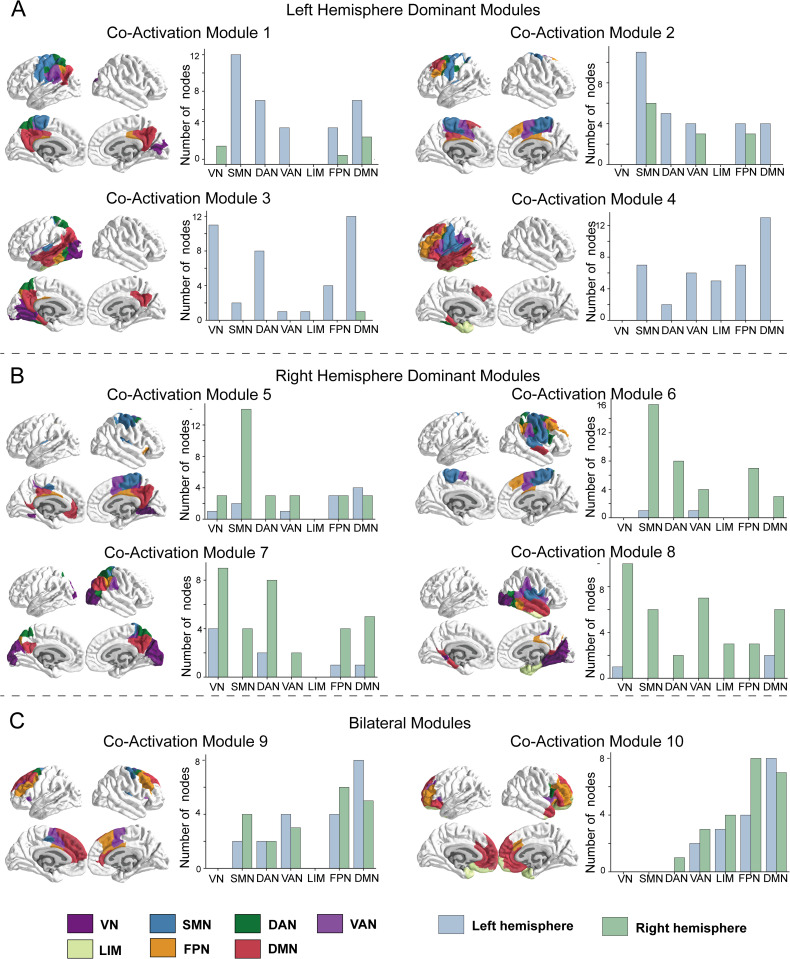
TMS-evoked cortical activity can be decomposed into 10 co-activation modules. These modules were summarized into 3 modes: (A) Left hemisphere dominant module, (B)Right hemisphere dominant module, and (C) Bilateral modules. Specifically, Module 1 and 2 mainly involve the left Somatomotor network (SMN) which can be regarded as the functional clusters of brain activity directly evoked by TMS. Meanwhile, module 5 and 6 mainly involve the right SMN which can be regarded as the contralateral modules to the stimulation modules. VN: Visual Network, DAN: Dorsal Attention Network, VAN: Ventral Attention Network, LIM: Limbic Network, FPN: Frontoparietal Network, DMN: Default Mode Network. All elements in this figure were created by the authors using hand-drawing and open-source Python software.

We identified 10 co-activation modules and investigate their spatial topology. Each node within these modules was assigned to one of 7 intrinsic functional network (ICNs): Visual Network (VN), Somatomotor Network (SMN), Dorsal Attention Network (DAN), Ventral Attention Network (VAN), Limbic Network (LIM), Frontoparietal Network (FPN), and Default Mode Network (DMN). To better interpret the spatial characteristics of the modules, we visualized the ICN affiliations of constituent brain regions within each module. As showed in Fig 2, the spatial distribution of these modules exhibited a high degree of centralization rather than dispersion, with pronounced hemispheric lateralization. We categorized these 10 modules into three primary patterns: ‘Left hemisphere dominant module’, ‘Right hemisphere dominant module’, and ‘Bilateral modules’. (1) Left hemisphere dominant module include module 1, 2, 3, 4. Modules 1 and 2 were predominantly associated with the left Somatomotor network (SMN), representing the primary clusters of brain activity directly evoked by TMS. (2) Right hemisphere dominant module include module 5, 6, 7, 8. Modules 5 and 6 involve the right SMN and were identified as contralateral counterparts to the left SMN stimulation modules. (3) Bilateral modules include module 9,10 which mainly involve both left and right DMN, FPN. The bilateral modules mainly involve high-level intrinsic functional networks. Moreover, we examined the structural and functional properties of co-activation modules by analyzing both structural and functional connections within each module. The dense structural and functional connections within the brain regions support the neural basis of co-activation modules. Please see Fig B in S1 Text for detailed results.

### Time-cumulative measure of TMS-evoked activity propagation

After decomposing the TMS-evoked activity into co-activation modules and corresponding time-varying weights, we next turn our attention to investigate the causal pathways of TMS-evoked activity propagation based on time-varying weights, which encode the temporal information. We calculated the Phase Slope Index (PSI) for the time-varying weights to estimate the direction of TMS-evoked activity propagation between different co-activation modules throughout the entire duration (0~400ms following stimulation) (Fig 1C). PSI is a mathematical method utilized to measure the information flow between multiple signals, and was extensively employed in fMRI, EEG and MEG to determine the directionality of activity propagation within the brain network, by evaluating the consistency of phase differences of functional activity between different brain regions over time [15].

**Fig 3:**
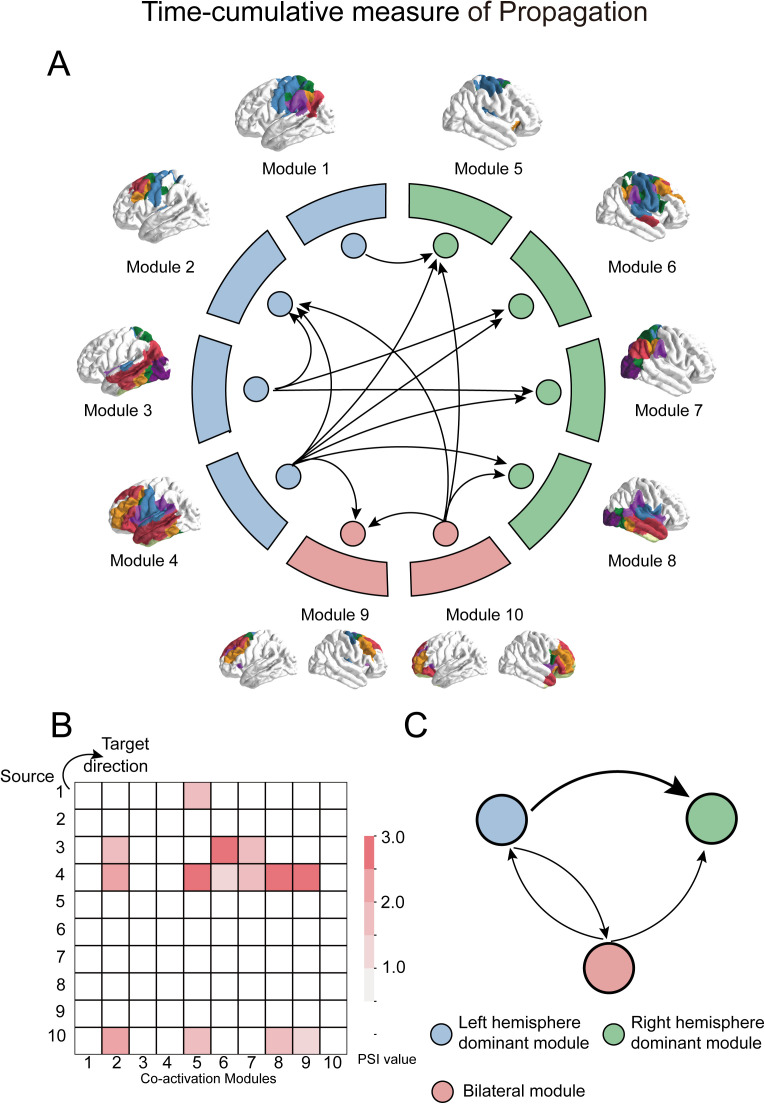
Time-cumulative measure of TMS-evoked activity propagation. This pattern implies that TMS-evoked activity extends beyond the directly stimulated regions, transmitting across the network to the contralateral brain regions. Panel A illustrates the directed propagation pattern, while panel B presents a PSI heatmap, where each cell indicates the PSI value from a source module (y-axis) to a target module (x-axis). Panel C illustrates the directed connectivity patterns among left hemisphere-dominant modules, right hemisphere-dominant modules, and bilateral modules. The thickness of each directed arrow reflects the summed strength of all directed connections from source to target module groups. For example, the arrow from the left hemisphere-dominant modules to the right hemisphere-dominant modules represents the total directed connectivity from modules 1, 2, 3, and 4 to modules 5, 6, 7, and 8. All elements in this figure were created by the authors using hand-drawing and open-source Python software.

The analysis provided a time-cumulative overview of the propagation patterns of TMS-evoked activity. As shown in Fig 3, we observed that left hemisphere-dominant modules, particularly Modules 1, 3, and 4, predominantly functioned as sources of propagation, with their output mainly directed toward right hemisphere-dominant modules (Modules 5–8), which in turn primarily acted as recipients. Bilateral modules (Modules 9 and 10) indicated distinct roles: Module 9 primarily received propagated signals, whereas Module 10 exhibited propagation toward Modules 2, 5, 8, and 9. Notably, Module 2, which corresponds to the directly stimulated brain region, might be expected to function primarily as a source of TMS-evoked activity propagation. However, the time-cumulative analysis revealed that Module 2 exhibited a high in-degree pattern, indicating that it predominantly received input from other modules. The term “in-degree” refers to the total strength of all incoming directed connections to a specific module, originating from graph theory. This counterintuitive observation suggests that the propagation of TMS-evoked activity may not be governed by a simple unidirectional flow, but could instead reflect a complex process unfolding over time, potentially involving multiple stages and dynamically evolving patterns of activity propagation. This highlights the importance of employing dynamic, stage-resolved analyses to accurately characterize the causal pathways of TMS-evoked responses within large-scale brain networks.

### Dynamic TMS-evoked activity propagation

To capture the dynamic propagation patterns of TMS-evoked brain signal, we adopted a hierarchical temporal framework that comprising fine-grained “states” and coarser-grained “propagation stages”. In this framework, states represent short, temporally stable configurations of cortical activity, while propagation stages integrate multiple adjacent states to reflect broader periods of activity propagation.

**Fig 4:**
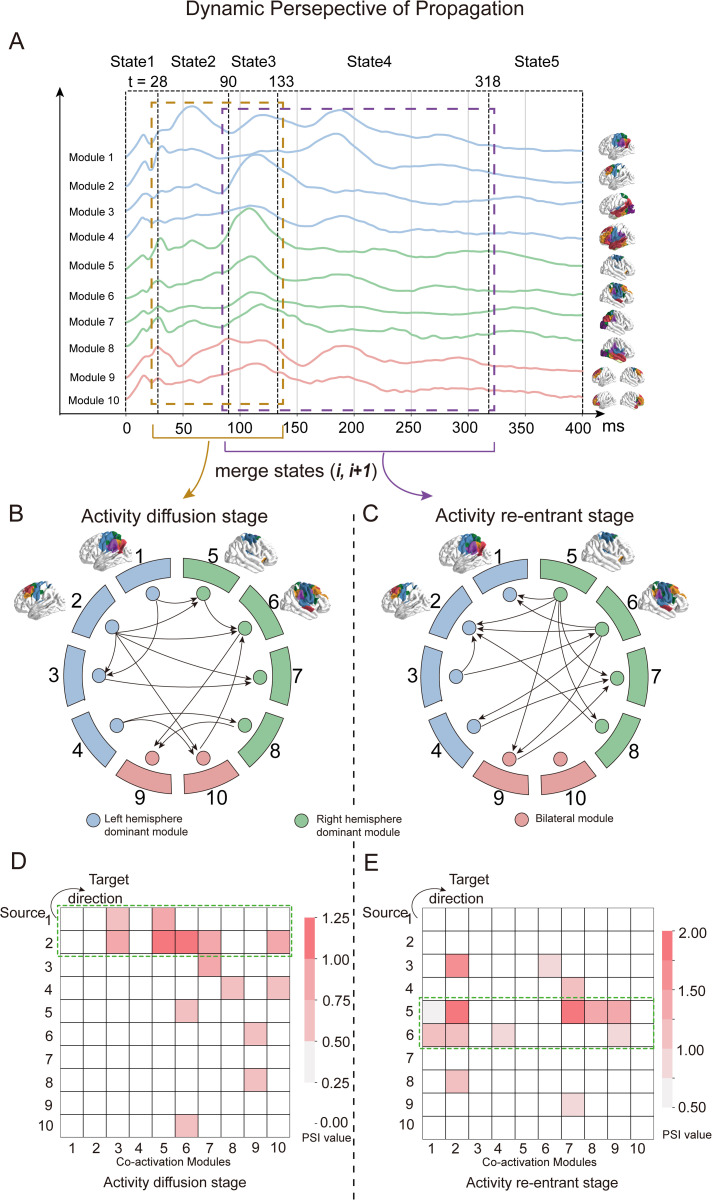
Stage-dependent PSI analysis revealed the dynamic TMS-evoked activity propagation. (A) The grand average TMS-evoked activity was partitioned into 5 states using the Greedy State Boundary Search (GSBS) algorithm. According to the first-order Markov assumption, we combined two adjacent states at a time to define propagation stages. Specifically, we merged states 2 and 3, and states 3 and 4 (corresponding to the Activity Diffusion Stage and Activity Re-entrant Stage, respectively), and calculated stage-dependent PSI for each (B, D) In the Activity Diffusion Stage, the propagation direction of TMS-evoked activity revealed that co-activation modules 1 and 2 acted as primary sources, propagating activity to both ipsilateral and contralateral brain regions. Panel B illustrates the directed propagation pattern, while panel D presents a PSI heatmap, where each cell indicates the PSI value from a source module (y-axis) to a target module (x-axis). (C, E) In the Activity Re-entrant Stage, co-activation modules 5 and 6 (contralateral to the stimulated region) served as sources, propagating activity back to the stimulated modules (1 and 2). Panel C shows the propagation direction, and panel E displays the corresponding PSI heatmap. All elements in this figure were created by the authors using hand-drawing and open-source Python software.

First, “state” was defined as a contiguous time segment in which cortical activity exhibits internal consistency and relative stability in its spatiotemporal pattern. We utilized the Greedy State Boundary Search (GSBS) algorithm, which uses a greedy optimization procedure to identify the location of state boundaries which partition the time series into multiple states [29]. The optimal number of states was determined by maximizing the *T*-distance, which quantifies the statistical separation between within-state similarity and between-consecutive-state similarity, thereby identifying the segmentation that best captures stable temporal patterns. In our data, GSBS was applied to the grand average time-varying weights and identified 5 optimal states spanning the 0–400 ms post-stimulation window. According to the peak of the time-varying weights, we examined these 5 states as follows (Fig 4A): (1) State 1 (1 ~ 28 ms) showed a nearly simultaneous increase in activity across all co-activation modules, triggering a very early peak, but far from reaching the global peak. However, this early response may be influenced by cranial muscle artifacts [30]; (2) State 2 (29 ~ 90 ms) indicated that the activity of co-activation modules 1 and 2 reach the global peak, while other modules maintain relatively stable. Notably, modules 1 and 2, as the directly stimulation targets, achieve their peaks first; (3) State 3 (91 ~ 133 ms) showed that the activity of almost all modules (except for modules 1 and 2) reaches the global peak, indicating the propagation of TMS-evoked activity throughout the whole brain during this state; (4) State 4 (134 ~ 318 ms) indicated that co-activation modules 1 and 2 reach the global peak again, while the activity of other modules remains relatively stable. We posit that TMS-evoked activity re-converges and propagates back to the directly stimulation targets; (5) State 5 indicates that the activity of all co-activation modules has decayed to approach baseline, indicating the end of the single-pulse TMS effect.

Second, Propagation stage was defined as temporally contiguous intervals formed by merging adjacent states to emphasize their role as physiologically meaningful periods of activity propagation. While each state reflects a temporally stable configuration, neural propagation likely unfolds across multiple such states due to causal or informational dependencies between them. According to the first-order Markov assumption which posits that the current state depends primarily on the immediately preceding one and not on more distant past states, we combine two adjacent states at a time to define a single propagation stage, as this preserves temporal dependency without overextending across multiple unrelated transitions. In this study, we excluded state 1, which exhibited global and nearly simultaneous activation possibly reflecting muscle artifacts, and state 5, where activity returned to baseline. We then focused on states 2, 3, and 4.

We defined stage-dependent PSI as the calculation of PSI within each propagation stage which merged adjacent states. We merged states 2 and 3 into a single propagation stage and then calculated the PSI of time-varying weights between co-activation modules. As showed in Fig 4B and 4D, a key pattern emerges wherein co-activation modules 1 and 2 serve as the source, propagating TMS-evoked activity to both ipsilateral and contralateral hemisphere brain regions. Based on this observed propagation pattern, we named this interval the “Activity Diffusion Stage”. In addition to this dominant pattern, activity propagation also occurred within the left hemisphere (from modules 1 or 2–3), within the right hemisphere (from module 5–6), and toward the bilateral module from both hemispheres. Similarly, we merged states 3 and 4 into another propagation stage and then calculated the PSI of time-varying weights between co-activation modules. As showed in Fig 4C and 4E, the most interesting discovery is that, co-activation modules 5 and 6 (contralateral to the stimulation brain regions), serve as the source, propagating the TMS-evoked activity back to the stimulation brain regions (modules 1 and 2). Based on this observed propagation pattern, we named this interval the “Activity Re-entrant Stage”. In addition to this dominant pattern, we also observed propagation within right-dominant modules (from module 5–7 and 8), within left-dominant modules (from module 3–2), and propagation to the bilateral module (module 9) from modules 5 and 6. In summary, from a dynamic perspective, the TMS-evoked activity propagation can be summarized as follows: Initially, TMS stimulation induces local activity in the directly stimulation brain regions (left M1). Next, TMS-evoked activity propagates throughout the whole brain, particularly to the contralateral hemisphere. Finally, TMS-evoked activity propagates back from the brain regions of contralateral hemisphere (right M1) to the stimulation brain region.

## Discussion

In the current study, we aimed to investigate the comprehensive propagation pathways of TMS-evoked brain responses in dynamic perspective. Utilizing the sparsity NMF method, the TMS-evoked activity was decomposed into low-dimensional combination of co-activation modules and corresponding time-varying weights which represents the spatial and temporal components respectively. Utilizing the stage-dependent effective connectivity method, we characterize the direction of TMS-evoked activity propagation in dynamic perspective. Our framework elucidated the comprehensive causal pathways of TMS-evoked responses: Firstly, single-pulse TMS induces local activity in the directly stimulated regions (left M1). Then, TMS-evoked activity propagates throughout the whole brain. Finally, the TMS-evoked activity propagates back from the brain regions of contralateral hemisphere (right M1) to the stimulation brain regions.

By using sNMF method, the TMS-evoked activity was decomposed into low-dimensional combination of co-activation modules and corresponding time-varying weights. our results revealed a pronounced hemisphere lateralization in the spatial distribution of co-activation modules during TMS-evoked activity. Notably, both the directly stimulated modules (modules 1 and 2) and the corresponding contralateral hemisphere modules (modules 5 and 6). These results suggested that the spatiotemporal structure of TMS-evoked brain activity was sensitively captured under the low-dimensional perspective. Moreover, dense structural and functional connections within the brain regions for each co-activation module (Fig B in S1 Text) also suggest that the modules have a robust neurobiological basis in the structural and functional networks.

The decision to decompose TMS-evoked activity into co-activation modules was motivated by two key reasons: (1) The flexible reconfiguration of modules reflects dynamics of brain network adapting to external stimuli and cognitive demands [31,32]. TMS-evoked brain activity is not randomly distributed across spatial scales; instead, it propagates through the brain networks, exhibiting spatial self-organization as it traverses different regions. Thus, we assume that TMS-evoked brain activity also exhibits co-activation modules at the spatial scale, and our results confirmed this. (2) Previous studies have indicated that NMF can effectively decompose dynamic brain activity or network into spatial and temporal components [28,33], enabling researchers to study the dynamic interactions of brain organization from a low-dimensional perspective, making complex brain activity more interpretable [26,32,34]. Applying NMF also enabled us to capture the spatiotemporal dynamics of TMS-evoked brain activity, providing a concise and interpretable perspective on activity propagation.

The key innovation of this study is the development of a novel framework that, by integrating intervention-based paradigms with dynamic effective connectivity analysis. Firstly, unlike traditional approaches relying on observational data such as resting-state recordings, our method leverages the power of concurrent TMS-EEG, which actively perturbs a targeted cortical region and measures its downstream effects in real time. This shift from passive observation to active manipulation enables more direct testing of causal hypotheses and enhances the validity of inferred directional influences between brain regions. Secondly, beyond leveraging the causal strength of TMS-EEG, we further proposed the analysis by introducing a hierarchical temporal framework to capture the dynamics of TMS-evoked brain activity. In previous dynamic connectivity analysis, particularly in resting-state studies, the sliding-window approach was widely used [35–37]. This method estimates functional or effective connectivity within fixed-length windows that move through the data with a defined step size. However, the choice of window length is often empirically driven, and more importantly, it fails to account for temporal dependencies between adjacent windows—neglecting the influence of one window on the next. These limitations reduce the accuracy of effective connectivity inference. To address these issues, we adopted a hierarchical temporal framework comprising fine-grained “states” and coarser-grained “propagation stages”, enabling more temporally informed characterization of dynamic neural propagation. Specifically, we used the Greedy State Boundary Search (GSBS) algorithm to identify temporally stable states of cortical activity. We then merged adjacent states to form propagation stages, motivated by the first-order Markov assumption that each state is primarily influenced by its immediate predecessor. This design preserves the natural temporal dependencies of brain activity and allows for stage-dependent estimation of effective connectivity.

By applying this framework, we identified two key propagation stages in TMS-evoked activity. In the diffusion stage (merging States 2 and 3), co-activation modules 1 and 2 emerged as primary sources, propagating activity across both hemispheres. In the subsequent re-entrant stage (merging States 3 and 4), modules 5 and 6—located contralateral to the stimulation site—acted as sources, relaying activity back to the original stimulation region. This pattern of dynamic propagation outlined a re-entry loop of dynamics, consistent with prior qualitative observations [25]. Moreover, our findings underscore the functional importance of contralateral regions in completing the re-entrant dynamics.

In clinical applications of repetitive TMS (rTMS) for neuropsychiatric disorders, once a stimulation target is determined, we propose incorporating concurrent TMS-EEG recordings to evaluate the target’s network-level influence. By applying our framework, one can identify the causal propagation pathways initiated from the selected target. This network-based characterization of stimulation effects may provide critical insights into the mechanisms underlying symptom improvement and help refine therapeutic strategies from a circuit-level perspective.

One of the most intriguing findings of our study is the discovery of the TMS-evoked re-entrant pathway. Re-entry in nervous systems is the important integrative mechanisms in vertebrate brains, which is characterized by the ongoing bidirectional exchange of signals along reciprocal axonal fibers linking multiple brain regions [38]. Through excitatory and inhibitory re-entrant connections, the brain establishes a distributed pattern of re-entrant activity which enables it can act to inhibit or compete with conflicting alternative response patterns [39,40]. Moreover, re-entrant activity is also believed to contribute to the spontaneous rhythmic activity by the mutual exchange of action potentials transmitted via reciprocal paths [41,42]. Previous studies suggested that re-entrant connections support interaction and synchronization between visual cortex, especially re-entrant projections from medial temporal area (MT) to primary visual cortex (V1) [43,44]. Further, by conducting cortico-cortical paired associative stimulation (ccPAS), which involves repeatedly pairs single-pulse TMS over V1 and MT regions, Bevilacqua et al. revealed a causal involvement of the re-entrant MT-to-V1 low-frequency inputs in motion discrimination and integration [23]. This finding also suggests that direction-specific network plasticity can be modulated, providing insight into how targeted stimulation of re-entrant connections may serve as an effective approach to regulating network plasticity. Overall, re-entrant dynamics facilitates functional integration between different cortical regions. our findings demonstrate the re-entrant dynamics in the primary motor cortex between left and right hemispheres which highlight the functional integration and communications between bilateral primary motor cortex.

Cortical activity propagation among separated brain regions is primarily mediated by networks of long-range tracts of myelinated axons that constitute white matter [38]. The corpus callosum is the principal white matter fiber bundle that connects homologous cortical areas of the two cerebral hemispheres [45,46], and plays a vital role in mediating interhemispheric communication, and integrates information across cerebral hemispheres [47]. Specifically, Previous anatomical studies in the rhesus monkey and imaging studies in human have found that callosal motor fibers connects the primary motor cortices in the two hemispheres [48]. Based on task-fMRI, studies have verified corpus callosum supports bidirectional communication in sensory, phonological, and cognitive processing [49]. A recent meta-analysis using simple visual integration paradigms compared interhemispheric neural integration in patients who had undergone complete commissurotomy, complete callosotomy, or partial callosotomy [50]. Cross-hemisphere visuo-motor integration was significantly slower in patients with complete callosotomy (43.5 ms) or full commissurotomy (60.6 ms), compared to those with partial callosotomy (8.8 ms) or healthy controls (2.86 ms). These findings provide strong evidence that the corpus callosum plays a critical role in facilitating electrophysiological signal transmission and integrating information across cerebral hemispheres.

## Limitations

The main limitation of our work is that the sensory-evoked response (the responses of shoulder and cranial muscle) may contaminate results. Specifically, the raw scalp response and sensory-evoked response exhibited correlation in both spatial and temporal domains, particularly after ~60 ms, irrespective of the intensity and stimulus waveform. To minimize the contribution of sensory-evoked response to the TMS-evoked potentials. Rogasch group utilized the state-of-art preprocessing pipeline getting the best trade-off between preserving the cortical activity and removing sensor-evoked signals, suggesting TMS-evoked potentials at least partly reflect the direct cortical response to TMS. Thus, based on this open data, we believe that our results are mainly driven by cortical response rather than sensory-evoked response. Secondly, Another limitation concerns the potential influence of volume conduction, which may introduce spurious correlations and reduce spatial specificity in EEG source localization. We addressed this by applying dSPM normalization, analyzing large-scale co-activation modules instead of ROI-level connections, and using the phase slope index (PSI), which is robust to volume-conducted mixtures [14]. Nevertheless, we acknowledge that volume conduction cannot be entirely eliminated. Thirdly, we found the dynamic, bidirectional propagation of TMS-evoked activity between the left and right hemispheres. However, this finding was based solely on stimulation target located on the left M1. Subsequent studies can validate this finding based on different stimulation targets. Last, in the dynamic analysis of TMS-evoked activity, we segmented different stages based on prior knowledge, and then calculated stage-dependent effective connectivity. Future work could develop data-driven methods for dynamic effective connectivity to more accurately infer dynamic causal pathways of TMS-evoked responses.

## Conclusion

In this study, we established a quantitative framework that investigate the comprehensive propagation pathways of TMS-evoked responses. We found that TMS induces local activity in the directly stimulated regions (left M1). Then, TMS-evoked activity propagates throughout the whole brain. Last, propagates back from the brain regions of contralateral hemisphere (right M1) to the stimulation brain regions. Overall, the propagation pathways of TMS-evoked brain responses can be summarized as a loop: ‘stimulation brain regions - contralateral brain regions - stimulations brain regions. This study provides a preliminary exploration of how local perturbation influence brain network dynamics, and offers insights into the neural mechanism of precise TMS intervention from a network perspective.

## Methods

### TMS-EEG data and source reconstruction

In this study, we used concurrent TMS-EEG dataset which was collected and provided to community by the Rogasch group (https://figshare.com/articles/dataset/TEPs-_SEPs/7440713, doi: https://doi.org/10.26180/5c0c8bf85eb24) under a CC BY-NC-ND 4.0 license. We used the dataset strictly for non-commercial research purposes and with appropriate attribution, in accordance with the license terms. The dataset consisted of a total of 20 healthy individuals (24.50 ± 4.86 years; 14 females), all of whom received single-pulse TMS stimulation on the left primary motor cortex (M1) while brain activity was recorded by density EEG [19]. TMS was applied over the left M1 using a figure-of-eight coil connected to a MagPro X100 stimulator. The coil was positioned tangentially to the scalp with the handle oriented at a 45° angle to the sagittal plane, and its location was guided using a neuronavigation system co-registered to each participant’s MRI. TMS intensity was set to 120% of each participant’s resting motor threshold, which was defined as the lowest stimulator output required to evoke motor-evoked potentials larger than 50 μV in at least 5 out of 10 trials.

The detailed description of the dataset and steps for state-of-art preprocessing can be found at the study of Biabani et al. [19]. Specifically, these preprocessing steps exhibited the best trade-off between preserving the cortical activity not related to the control condition and removing sensor-specific signals. Next, we performed source reconstruction by using dSPM method based on MNE software library. The outcome of EEG source reconstruction yielded a time series of dSPM current densities at each cortical surface location. Finally, the time series were extracted through Schaefer 200 parcellations atlas [51].

### Sparse nonnegative matrix factorization

We employed sparse nonnegative matrix factorization (sNMF) method to decompose the source-dependent trial-average TMS-evoked activity of each subject into co-activation modules and time-varying weights [26,52]. The co-activation modules effectively capture spatial information, offering insights into the spatial distribution of TMS-evoked co-activation activity, while the time-varying weights encode temporal information, reflecting the activation strength of modules over time. Mathematically, the linear combination of co-activation modules and time-varying weights can approximately reconstruct the TMS-evoked activity. Moreover, we conducted sparse optimization on co-activation modules, highlighting the most active components. In our formula, we defined the concatenation of the source-dependent TMS-evoked activity of input subjects as $X\in R^{P\times N}$, where *P* represented the number of brain regions (200 in our study), and *N* represented the number of time points of input subjects. The optimization function was as follows:


$${arg}_{U,V}min{\left\|X-U\times V\right\|}_{F}^{2}\;$$ (1)

$$subject\;to\;{\;\left\|u_{r}\right\|}_{0}\leq \alpha \cdot P$$

$$0\leq u_{r}(i)\leq 1,\;max_{i}\left|u_{r}(i)\right|=1\;$$

V≥0

Where $U=\left[u_{1},\;...,u_{R}\right]\in R^{P\times R}$represented the concatenation of each co-activation module, each $u_{r}\in R^{P\times 1}$ represented the spatial pattern of the *r*th co-activation module, and *R* represented the number of co-activation module (R≪P). While $V=\left[v_{1},\;...,v_{R}\right]\in R^{R\times P}$ was the concatenation of corresponding time-varying weights, and the vector $v_{r}\in R^{N\times 1}$ was the temporal expression of *r*th co-activation module of all time points. The objective function $min{\left\|X-U\times V\right\|}_{F}^{2}$ aimed to minimize the reconstruction error between TMS-evoked activity X and the linear combination of co-activation module U and time-varying weights V. Importantly, the term ${\;\left\|u_{r}\right\|}_{0}\leq \alpha \cdot P$ represented the spatial sparsity constraint on the co-activation modules. The L0 norm on $u_{r}$, represented as ${\left\|u_{r}\right\|}_{0}$, was employed as a sparsity regularization term that tends to favor solutions with fewer non-zero elements, thereby facilitating sparse representation. α was the sparsity ratio (0<α<1), and the term α·P was the maximal number of non-zero coefficients for each co-activation module (per column of U). The introduction of sparsity in the NMF method serves a crucial role in enhancing the interpretability and specificity of the identified co-activation modules. By setting certain components to zero, the sparsity constraint effectively highlights the most dominant spatial patterns of TMS-evoked activity while suppressing low-amplitude signals and potential noise. This approach ensures that the resulting modules capture the most functionally relevant and distinct network activity [53]. Furthermore, we normalized $u_{r}$, such that $0\leq u_{r}(i)\leq 1\;{and\;max}_{i}\left|u_{r}(i)\right|=1$ to make time-varying weights $v_{r}$ to be comparable.

To obtain the optimal variable U (co-activation module) and V (time-varying weights), we applied the alternating minimization strategy to solve this problem: (1) we randomly initialized the variable U and V. (2) At each iteration, we fixed V to optimize U by using nonnegative orthogonal matching pursuit algorithm, while imposing sparsity (${\;\left\|u_{r}\right\|}_{0}\leq \alpha \cdot P$) and normalization constraints ($0\leq u_{r}(i)\leq 1\;{and\;max}_{i}\left|u_{r}(i)\right|=1$). Then we fixed U to optimize V by using the gradient descent algorithm until fit ceases to improve. (3) Repeat until the fit ceases to improve or the maximum number of iterations is reached.

The free parameters of sNMF model were the number of co-activation modules *R* and L0-norm parameter α. Determining the appropriate free parameters plays a crucial role in the optimization of variable U and V. We implemented a cross-validation procedure to assess the stability of the decomposition and select optimal parameters. (1) We split the trial-average TMS-evoked EEG data from all 20 subjects into two non-overlapping and equally sized subsets, each comprising data from 10 participants. (2) Applying a grid search approach, each combination of parameters α∈[10%,15%,20%...50%] and R∈[6,7,8...12] was utilized to train the sNMF model on the both 2 subsets, subsequently yielding the reconstruction error. The reconstruction error was computed as the Frobenius norm of the residual matrix between the original and reconstructed TMS-evoked EEG data, normalized by the Frobenius norm of the original EEG matrix. This yields a percentage measure, where values closer to 0 indicate better reconstruction quality and values approaching 100% reflect higher error. The reconstruction error was defined as follow:


$$R_{res}\;=\;\frac{{\left\|X-U\times V\right\|}_{F}^{2}}{{\left\|X\right\|}_{F}^{2}}\;$$ (2)

(3) Our goal in optimizing the sNMF parameters was to balance two key objectives: achieving stable co-activation modules across independent subject groups, and minimizing reconstruction error, which reflects the model’s goodness of fit. We found that increasing the number of components (*R*) or reducing sparsity (using higher *α* values) generally decreased the reconstruction error (Fig A in S1 Text), indicating better data fitting. However, these settings did not consistently produce stable decompositions, likely due to the inclusion of redundant or noise-driven components. To address this trade-off, we selected the parameter configuration that best balanced both criteria. Specifically, R = 10 and α = 20% yielded the highest inter-group module stability (Pearson correlation = 0.64) while maintaining a low reconstruction error ($R_{res}$ = 25%), suggesting this combination offers an optimal compromise between model accuracy and reproducibility.

### Phase slope index

Considering that time-varying weights encode temporal information, we calculated the phase slope index (PSI) for the grand average time-varying weights to investigate the direction of TMS-evoked activity propagation between co-activation modules. Here, the grand average refers to averaging across subjects, rather than across modules or time. This group-level averaging enhances the signal-to-noise ratio and enables the extraction of stable and reproducible temporal dynamics, minimizing the influence of individual variability. The core concept of PSI is rooted in the principle that when one brain region transmits information to another, the signal transmission incurs a delay due to finite conduction time. This delay results in a phase lag between the sender and receiver signals, and critically, this lag increases systematically with frequency. PSI detects this characteristic pattern: if the phase difference between two signals increases with frequency, it indicates a consistent time delay, suggesting that one region is causally influencing the other. this frequency-dependent phase shift reflects a constant transmission delay, leading to a linear increase in phase difference with frequency. By quantifying this slope, PSI provides a robust estimate of the directionality of information flow [15]. The PSI ${\tilde{\Psi }}_{i,j}$ between different co-activation modules was defined as:


$${\tilde{\Psi }}_{i,j}=I\left(\sum_{f\in F}C_{i,j}^{*}(f)C_{i,j}(f+\delta f)\right)\;$$ (3)

Where: (1) ℑ(·) denoted the imaginary part of the complex quantity; (2) F was the set of discrete frequencies within the analysis band; in our study, we define **F={f:1* ≤ f ≤ 100} Hz*, which matches the preprocessing bandpass filter range used in Biabani et al.[19]; (3) δf was the frequency resolution, defined by the spacing between adjacent frequency bins; (4) “*” indicated the complex conjugate; (5) $C_{i,j}(f)$ was the complex coherency of time-varying weights between modules *i* and *j* at frequency f, and was calculated as:


$$C_{i,j}(f)=S_{i,j}(f)/\sqrt{S_{i,i}(f)S_{j,j}(f)}\;$$ (4)

Where $S_{i,j}(f)$ was the cross-spectral density of time-varying weights between modules *i* and *j*, which was calculated as:


$$S_{i,j}(f)=\left\langle {\tilde{v}}_{i}(f){\tilde{v}}_{j}^{*}(f)\right\rangle \;$$ (5)

with ${\tilde{v}}_{i}(f)$ denoting the Fourier transform of the time-varying weights of module *i*, and ${\tilde{v}}_{j}^{*}(f)$ denoting its complex conjugate. To remove potential spurious connections and emphasize the important ones, we thresholded PSI matrix by reserving the top 30% of connections (${\tilde{\Psi }}_{i,j}$ higher than threshold) and removing 70% weak connections which was commonly adopted in graph-theoretical studies of brain networks [54,55].

### State segmentation

A state is defined as a contiguous time segment in which cortical activity exhibits internal consistency and relative stability in its spatiotemporal pattern. To identify these states, we employed the Greedy State Boundary Search (GSBS) algorithm, which uses a greedy optimization procedure to identify the location of state boundaries which partition time-varying weights into multiple time segments [29].

GSBS is conducted by an iterative framework: (1) Initialization: All time points are initially assigned to a same state. (2) Boundary Candidate Evaluation: In each iteration, every time point is considered as a potential boundary location for splitting an existing state into two states. For each candidate boundary, the algorithm computes the mean activity pattern (called state template) for both resulting states. Then, for each time point, the algorithm calculates the Pearson correlation between the original activity pattern and the corresponding state template. The average of these correlations across all time points yields a fit score for the candidate boundary and the associated state assignment. (3) Greedy Selection of Boundary: The boundary that produces the highest fit score is selected, and the current state is split at that location to create two new states. This process maximizes internal similarity (within-state consistency) and ensures that each new state reflects a distinct temporal configuration of the cortical activity. (4) finding the optimal numbers of states: The iterative process continues until a maximum number of states is reached. To determine the optimal number of states, the GSBS framework employs a *T*-distance metric. This metric quantifies the statistical distance between within-state similarity and between-consecutive-state similarity. The number of states that maximizes the *T*-distance is selected as optimal, ensuring both high within-state coherence and clear transitions between states.

To compute the *T*-distance for a given number of states *k,* the first step is to calculate the pairwise correlation between cortical activity patterns at each pair of time points *i, j* as:


$$\rho =corr\left(x_{i},x_{j}\right)\;:\;i\neq j\;$$ (6)

Pairs of time points belonging to the same state contribute to the within-state correlation distribution $\rho_{w}(k)$, while pairs belonging to consecutive states contribute to the between-consecutive-state correlation distribution $\rho_{b}(k)$. The degree of separation between these two distributions is quantified using a t-statistic:


$$t_{\rho_{w}(k),\rho_{b}(k)}=\frac{{\hat{\rho }}_{w}(k)-{\hat{\rho }}_{b}(k)}{\sqrt{\frac{Var(\rho_{w}(k))}{N_{w}}+\frac{Var(\rho_{b}(k))}{N_{b}}}}\;$$ (7)

Where $N_{w}$ is the number of within state correlation values and $N_{b}$ is the number of between-consecutive-state correlation values. The *T*-distance is calculated for each candidate value of *k*, and the number of states that yields the maximum *T*-distance is selected as the optimal segmentation. In our study, the segmentation into 5 temporal states was not arbitrarily chosen but determined using the GSBS algorithm combined with the *T*-distance metric. This approach identified five states as the optimal number, maximizing the separation between within-state and between-state correlation distributions.

### Stage-dependent PSI

To investigate the directionality of activity propagation over time, we refer “propagation stages” to emphasize their role as physiologically meaningful periods of activity propagation that are constructed by combining multiple adjacent states. The rationale for grouping adjacent states into propagation stages is grounded in two considerations. (1) Each state, identified by the GSBS algorithm, captures a temporally stable configuration of TMS-evoked activity. However, activity propagation is a complex process that typically unfolds across multiple such states rather than being confined to a single, stable configuration. (2) Adjacent states are not independent of one another; rather, there is likely a causal or informational influence from one to the next. In this context, a propagation process is not confined within a single state but may extend across multiple neighboring states. According to the first-order Markov assumption which posits that the current state depends primarily on the immediately preceding one and not on more distant past states, we combine two adjacent states at a time to define a single propagation stage, as this preserves temporal dependency without overextending across multiple unrelated transitions.

We defined stage-dependent PSI as the calculation of PSI within each propagation stage. Consistent with the previous section, we reserved the top 30% of connections in the PSI matrix. Given the characteristics of states 1 and 5—state 1 likely reflecting early non-neural artifacts, and state 5 representing the post-response decay, we focused our analysis on states 2, 3, and 4, which reflect the core propagation dynamics. We merged states 2 and 3 to define one propagation stage, and states 3 and 4 to define a second propagation stage. We subsequently computed the PSI between co-activation modules within each stage to determine the directionality of activity propagation.

## Supporting information

S1 Text S1 Text contains further details about the results. This file includes Figs A to E and Table A. **Fig A:** Optimization results for two key parameters in the model. **Fig B**: The dense structural and functional connections within the brain regions support the neural basis of co-activation modules. **Fig C:** The heatmap of phase slope index (PSI) matrix (Not thresholding) represents the direction of TMS-evoked activity propagation between different co-activation modules throughout the entire duration. **Fig D:** The heatmap of PSI matrix (***Not thresholding***) during activity diffusion stage and activity re-entrant stage. **Fig E:** The heatmap of PSI matrix which was calculated within each individual state. **Table A:** The statistic result of shortest path length of structural connectivity and average functional connectivity in each co-activation module.
(DOCX)
