# Supplementary material for: Tracking causal pathways in TMS-evoked brain responses
Source: PLoS Comput Biol. 2025 Jul 28;21(7):e1013316. doi: 10.1371/journal.pcbi.1013316 (PMC12313060; doi:10.1371/journal.pcbi.1013316)
Supplement: S1 Text — This file includes Figs A to E and Table A. Fig A: Optimization results for two key parameters in the model. Fig B: The dense structural and functional connections within the brain regions support the neural basis of co-activation modules. Fig C: The heatmap of phase slope index (PSI) matrix (Not thresholding) represents the direction of TMS-evoked activity propagation between different co-activation modules throughout the entire duration. Fig D: The heatmap of PSI matrix (Not thresholding) during activity diffusion stage and activity re-entrant stage. Fig E: The heatmap of PSI matrix which was calculated within each individual state. Table A: The statistic result of shortest path length of structural connectivity and average functional connectivity in each co-activation module. (DOCX) [file pcbi.1013316.s001.docx]

**Supplementary Information**

***EEG Preprocessing***

The pipeline for cleaning and analyzing TMS-EEG data was implemented using the EEGLAB and TESA toolboxes. First, EEG signals were concatenated and epoched from -1000 ms to 1000 ms around the TMS pulse, with baseline correction applied using the -500 to -10 ms window. The TMS pulse artifact (-2 to 15 ms) was removed and replaced via cubic interpolation, and data were downsampled to 1000 Hz. Trials and channels with prominent artifacts were visually inspected and removed. TMS-induced muscle and decay artifacts were identified and rejected using FastICA (1), followed by linear interpolation of missing data. The signals were then band-pass filtered (1–100 Hz) and band-stop filtered (48–52 Hz) with a zero-phase Butterworth filter (order = 4). Additional artifacts, such as eye blinks and noise, were corrected with a second FastICA run, with components selected via TESA automated functions and confirmed by visual inspection. Rejected channels were spatially interpolated using the spherical method, and all data were re-referenced to the common average. TMS-evoked potentials (TEPs) and peripherally-evoked potentials (PEPs) were obtained by averaging the recordings over trials for each participant and condition. Finally, signal-space projection with source-informed reconstruction (SSP-SIR) method was applied to suppress the contribution of PEPs from TEPs. Notably, Biabani et al demonstrated that SSP-SIR showed the best trade-off between removing sensory-related signals while preserving data not related to the control condition (2).

***Parameters selection***

The free parameters of sNMF model are the number of co-activation modules *R* and L0-norm parameter $\alpha$. Firstly, we split the trail-average TMS-evoked brain activity of all subjects (20 subjects) into 2 subsets; each subset contained TMS-evoked brain activity of 10 subjects. Then, by applying a grid search approach, each combination of parameters $\alpha\in[10\%,15\%,20\%...50\%]$ and $R\in[6,7...12]$ was utilized to train the sNMF model on the both 2 subsets. We hypothesized that the co-activation modules $U$ should exhibit relative stability across different subjects. Therefore, we calculated the similarity of $U$ between 2 subsets under the same parameter combination. As showed in **Fig A**, our results indicated stable solutions at $\alpha=20\%$ and $R=10$. Moreover, we calculated the reconstruct error for each parameter combination. The reconstruction error was defined as follow:

$$error = \frac{\left\| X-U\times V \right\|_{F}^{2}}{\left\| X \right\|_{F}^{2}} (1)$$

$X\in R^{P\times N}$ represents source-level TMS-evoked activity, $U\in R^{P\times R}$ represents the concatenation of each co-activation module, and $V\in R^{R\times P}$ represents time-varying weights. As showed in Fig A, panel B, the result indicated that as $\alpha$ and $R$ increase, the reconstruction error decrease. Additionally, it was found that when $\alpha$ exceeds 20%, the reconstruction error remains relatively stable. Considering the balance between stability and reconstruction error, we chosen $\alpha=20\%$ and $R=10$ as the optimal parameters.


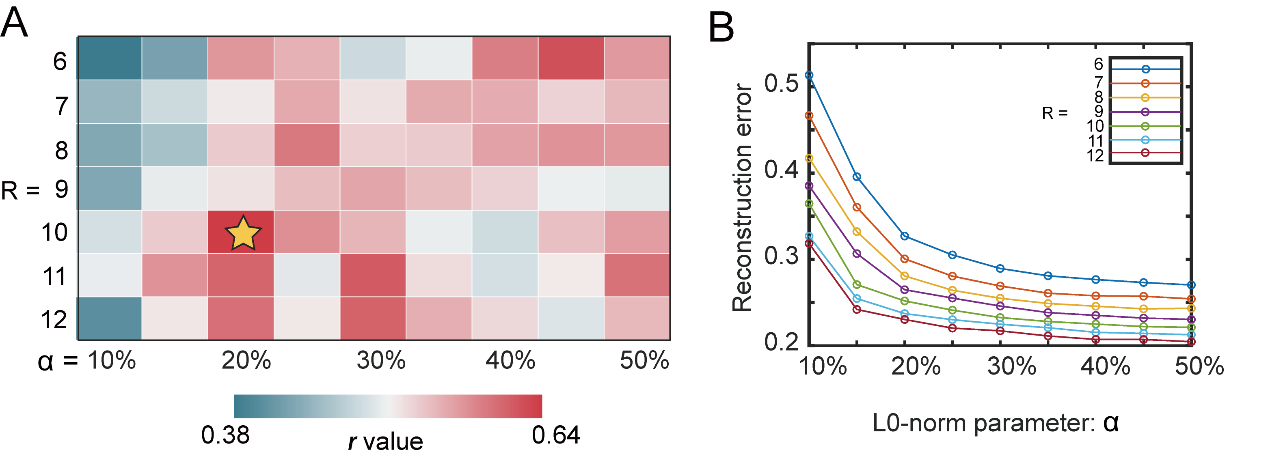


Fig A. Optimization results for 2 key parameters in our model. (A) the correlation coefficient of co-activation between 2 subsets under the same parameter combination. (B) the reconstruction error under each parameter combination.

***Linked co-activation modules to structural connectivity, functional connectivity, and neurotransmitter receptors***

We explored the structural and functional properties of co-activation modules by analyzing both structural and functional connections within each module.

For structure connectivity analysis, we utilized a healthy subject population-representative structural connectome matrix. This matrix comes from Human Connectome Project (HCP) which consist of structural neuroimaging data of 400 healthy young individuals (170 males; age range 21-35 years), and was calculated by (3). This matrix can be available at <https://github.com/GriffithsLab/PyTepFit>. Firstly, the structural connectivity matrix was binarized by using top 10% threshold. Then, the shortest path length between each pair of brain regions was calculated, and the shortest path length within co-activation module was averaged. Finally, the permutation test was conducted to verify whether the shortest path length within each module was significantly lower than random levels. Our results showed that the shortest path length nearly of all modules (except module 5) was significantly lower than random levels (**Table A and Fig B, panel A**), which indicated that structural connections within each module exhibit high network efficiency.

For functional connectivity analysis, we extracted group-level functional connectivity matrix from BrainSpace toolbox which was derived from HCP S900 dataset (4, 5). The averaged functional connectivity of each module was calculated, and the permutation test was conducted to verify whether the functional connectivity strength within each module was higher than random levels. Our results showed functional connectivity strength of nearly most modules (except module 4, 9 and 10) was significantly higher than random levels (**Table A, Fig B, panel B**).

TMS-evoked potentials are resulted from time-varying summation of excitatory and inhibitory postsynaptic potentials which are closely associated with neurotransmitter receptors system (6). Recently, the neurotransmitter receptor density maps, which were estimated using PET tracer studies for a total of 19 receptors and transporters, across 9 neurotransmitter systems, and including 1200 individuals, were available by Hansen and colleagues (7). These include serotonin (5-HT1A, 5-HT1B, 5-HT2A, 5-HT4, 5-HT6, and 5-HTT), glutamate (mGluR5 and NMDA), dopamine (D1, D2, and DAT), noradrenaline (NAT), GABA (GABA_A_), histamine (H3), acetylcholine (α4β2, M1, and VAChT), cannabinoid (CB1), and opioid (MOR). Based on these datasets, we sought to link co-activation modules to cortical patterning of neurotransmitter receptors. A multiple linear regression model was built which fits the neurotransmitter density maps from co-activation modules. The regression models including D1 ($R^{2}=0.51, p=0.004$), DAT ($R^{2}=0.49, p=0.029$), M1 ($R^{2}=0.25, p=0.036$), NMDA ($R^{2}=0.25, p=0.007$), and VAChT ($R^{2}=0.34, p=0.013$), were significant which assessed against a spin-permuted null model (10000 repetitions). As showed in Fig B, panel C, we conducted a dominance analysis to identified modules that contributed most to fit, by assigning a proportion of the $R^{2}$ to each variable to significant models.


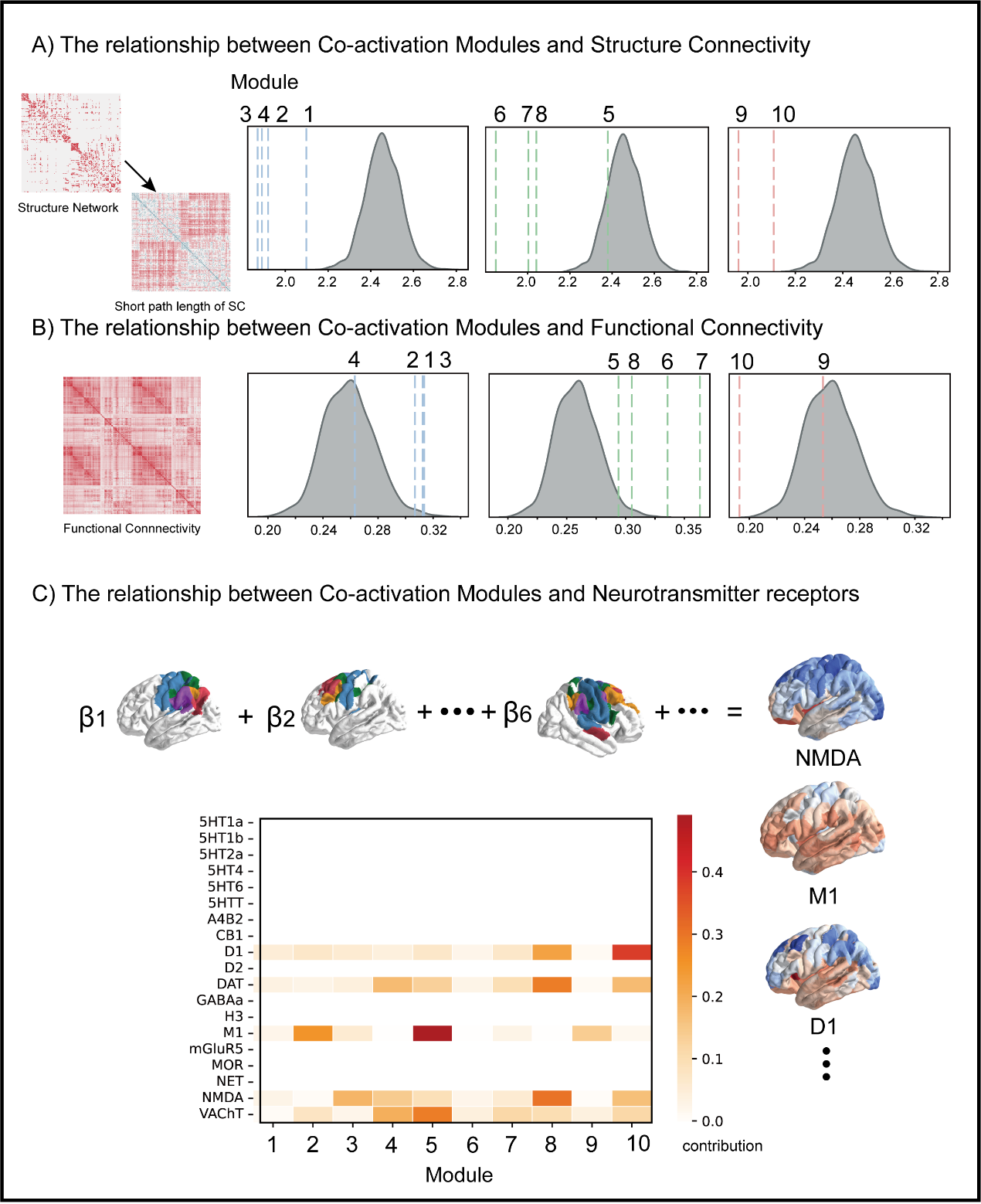


Fig B. (A) The shortest path length of structural connectivity in each co-activation module was calculated, and nearly of all modules (except module 5) was significantly lower than random levels. (B) The functional connectivity strength in each co-activation module was calculated, and nearly most modules (except module 4, 9 and 10) was significantly higher than random levels. (C) The multiple linear regression model was built which fits the neurotransmitter density maps from co-activation modules. The regression models including D1, DAT, M1, NMDA and VAChT were significant which assessed against a spin-permuted null model. All elements in this figure were created by the authors using hand-drawing and open-source Python software.

|  | Shortest path length | *P_perm_* | Average FC strength | *P_perm_* |
| --- | --- | --- | --- | --- |
| Module 1 | 2.10 | <0.001 | 0.31 | 0.004 |
| Module 2 | 1.92 | <0.001 | 0.31 | 0.01 |
| Module 3 | 1.87 | <0.001 | 0.31 | 0.004 |
| Module 4 | 1.89 | <0.001 | 0.26 | 0.39 |
| Module 5 | 2.38 | 0.1570 | 0.29 | 0.03 |
| Module 6 | 1.85 | <0.001 | 0.34 | <0.001 |
| Module 7 | 2.00 | <0.001 | 0.36 | <0.001 |
| Module 8 | 2.04 | <0.001 | 0.31 | 0.01 |
| Module 9 | 1.96 | <0.001 | 0.25 | 0.60 |
| Module 10 | 2.10 | <0.001 | 0.19 | 1 |

Table A. The statistic result of shortest path length of structural connectivity and average functional connectivity in each co-activation module.

***Time-cumulative measure of TMS-evoked activity propagation***


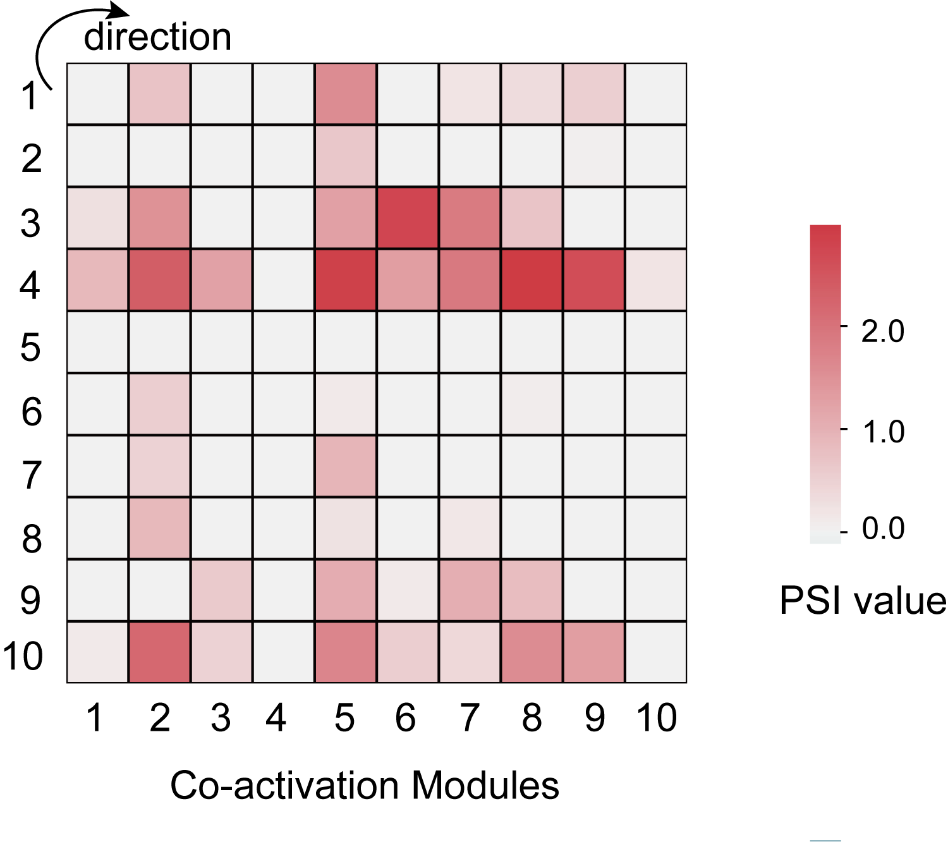


Fig C. The heatmap of phase slope index (PSI) matrix (***Not thresholding***) represents the direction of TMS-evoked activity propagation between different co-activation modules throughout the entire duration. The PSI matrix is unthresholded and retains only the positive connections between each pair of modules. Positive connections in PSI indicates that the first signal is leading the second signal, potentially suggesting a causal or a time-delayed interaction.

***Dynamic analysis of TMS-evoked activity propagation***


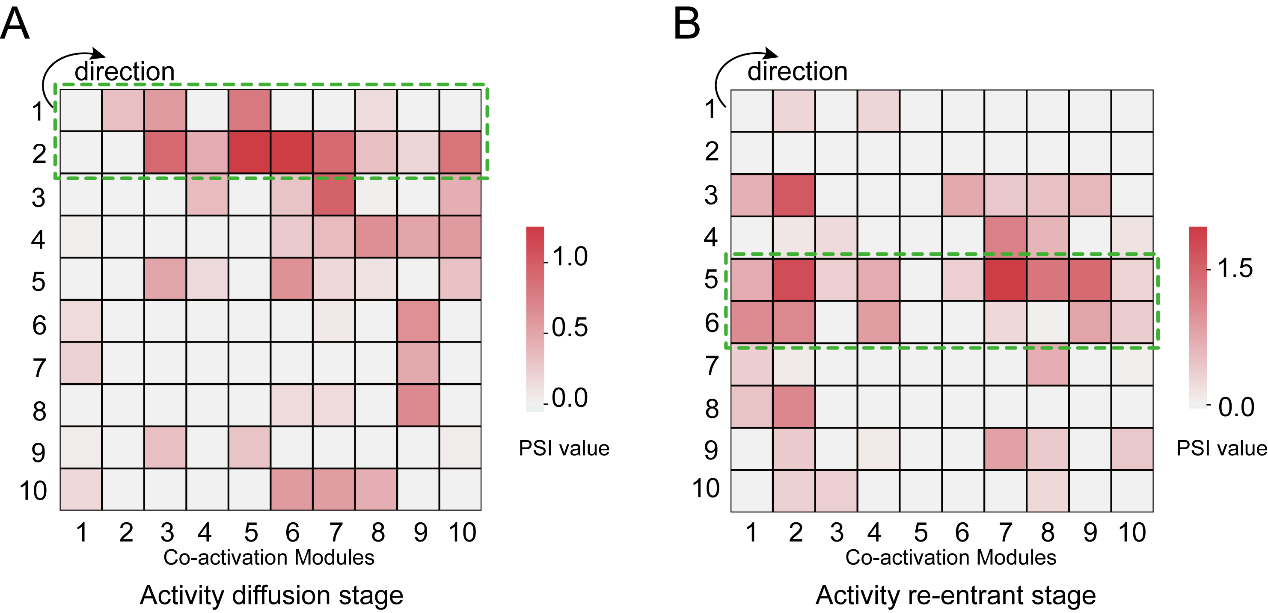


Fig D. (A) The heatmap of PSI matrix (***Not thresholding***) during activity diffusion stage (combined stage 2 and 3). A key pattern emerges wherein co-activation modules 1 and 2 serve as the source for signal transmission, propagating TMS-evoked activity to both ipsilateral and contralateral hemisphere brain regions. (B) The heatmap of PSI matrix (***Not thresholding***) during activity re-entrant stage (combined stage 3 and 4). co-activation modules 5 and 6 (contralateral to the stimulation brain regions), serve as the source for signal transmission, propagating the TMS-evoked activity back to the stimulation brain regions (modules 1 and 2). For both A and B, positive connections in PSI indicates that the first signal is leading the second signal, potentially suggesting a causal or a time-delayed interaction.

***State-wise PSI***

We computed PSI matrices for all five states and visualized them using heatmaps. While States 2, 3, and 4 reflect core propagation periods (State 1 may reflect muscle artifacts, and State 5 likely represents a return to baseline activity), their PSI matrices show difficult-to-interpret patterns: (1) In State 2, modules 4 acts as sources, propagating activity to other modules, and modules 1 and 2 receive input from Modules 3 and 4; (2) In State 3, modules 1, 2, and 10 appear primarily as targets, receiving input from other module; (3) In State 4, modules 3 and 8 act as sources , propagating activity to other modules. Notably, modules 1 and 2 (directly stimulated regions) consistently receive input from modules 3 and 4 across nearly all states, which is counterintuitive. Furthermore, module 3 (left temporal regions), unexpectedly serves as a dominant source across multiple states, a pattern difficult to reconcile with known neuroanatomy or stimulation protocols. When considering States 2–4 collectively, we were still unable to construct a coherent and physiologically plausible propagation trajectory. These inconsistencies suggest that PSI computed at the single-state level fails to capture the temporally integrated nature of causal propagation.


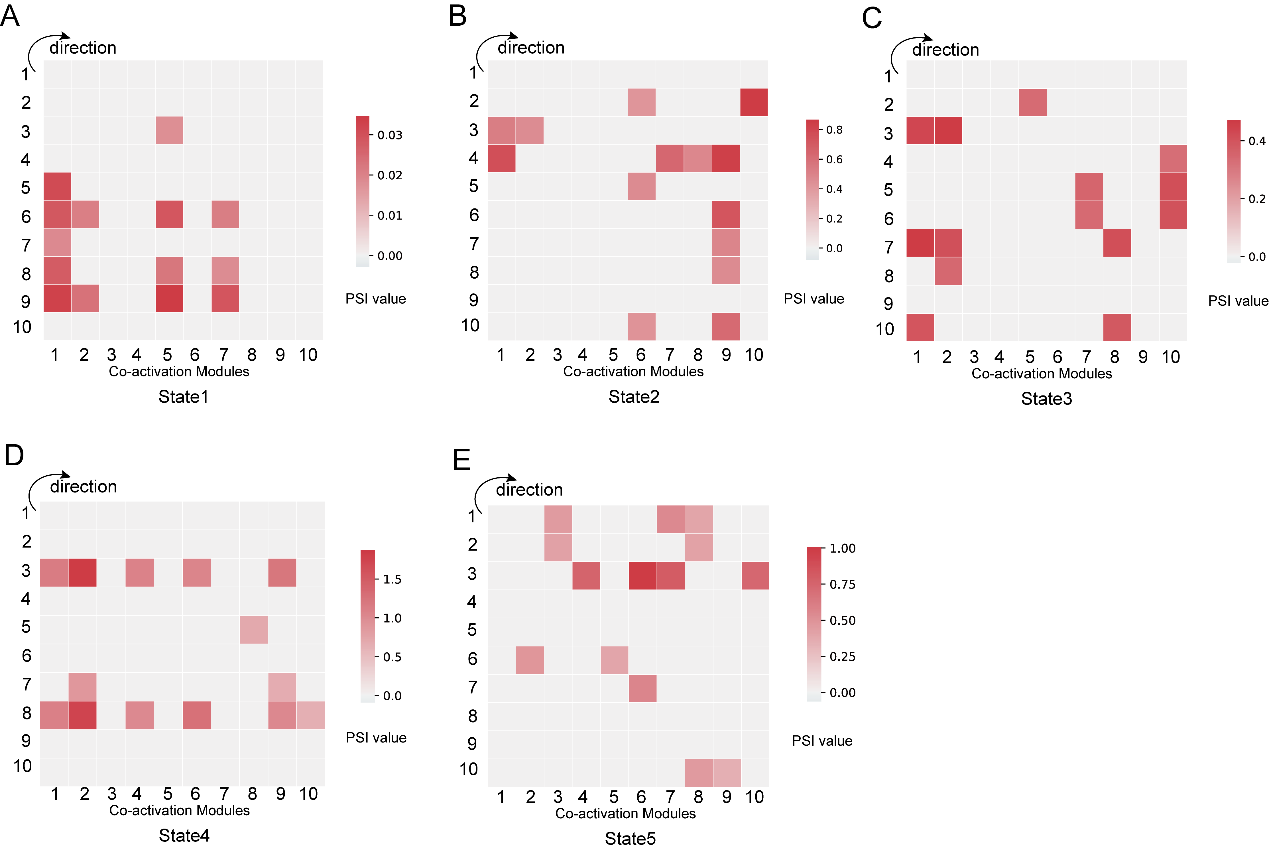


Fig E. The heatmap of PSI matrix which was calculated within each individual state. each cell indicates the PSI value from a source module (y-axis) to a target module (x-axis), reflecting directed information flow specific to that state

**References**

1. Hyvärinen A, Oja E. Independent component analysis:: algorithms and applications. Neural Networks. 2000;13(4-5):411-30.

2. Biabani M, Fornito A, Mutanen TP, Morrow J, Rogasch NC. Characterizing and minimizing the contribution of sensory inputs to TMS-evoked potentials. Brain Stimulation. 2019;12(6):1537-52.

3. Momi D, Wang Z, Griffiths JD. TMS-evoked responses are driven by recurrent large-scale network dynamics. Elife. 2023;12.

4. de Wael RV, Larivière S, Caldairou B, Hong SJ, Margulies DS, Jefferies E, et al. Anatomical and microstructural determinants of hippocampal subfield functional connectome embedding. Proceedings of the National Academy of Sciences of the United States of America. 2018;115(40):10154-9.

5. de Wael RV, Benkarim O, Paquola C, Lariviere S, Royer J, Tavakol S, et al. BrainSpace: a toolbox for the analysis of macroscale gradients in neuroimaging and connectomics datasets. Communications Biology. 2020;3(1).

6. Darmani G, Ziemann U. Pharmacophysiology of TMS-evoked EEG potentials: A mini-review. Brain Stimulation. 2019;12(3):829-31.

7. Hansen JY, Shafiei G, Markello RD, Smart K, Cox SML, Norgaard M, et al. Mapping neurotransmitter systems to the structural and functional organization of the human neocortex. Nature Neuroscience. 2022;25(11):1569-+.
